# Clinical practice of continuous rhythm monitoring after embolic stroke of undetermined source

**DOI:** 10.1371/journal.pone.0302404

**Published:** 2024-04-17

**Authors:** Aenne Solvejg von Falkenhausen, Johannes Wischmann, Linus M. Keidel, Antonia M. Kellnar, Raffael Thaler, Korbinian Lackermair, Heidi L. Estner, Günter Höglinger, Steffen Massberg, Stefan Kääb, Lars Kellert, Moritz F. Sinner

**Affiliations:** 1 Department of Medicine I, LMU University Hospital, LMU Munich, Munich, Germany; 2 German Centre for Cardiovascular Research (DZHK), Partner Site: Munich Heart Alliance, Munich, Germany; 3 Department of Neurology, LMU University Hospital, LMU Munich, Munich, Germany; Baruch Padeh Medical Center Poriya, ISRAEL

## Abstract

**Aims:**

Embolic stroke of undetermined source (ESUS) accounts for up to 20% of ischemic strokes annually. Undetected atrial fibrillation (AF) is one important potential underlying cause. For AF, oral anticoagulation has evolved as the most preferable means of secondary stroke prevention. To detect unrecognized paroxysmal AF, long-term ECG monitoring is required, and implantable cardiac monitors (ICM) appear most suitable. Yet, ICMs are particularly costly, implantation is invasive, and remote monitoring places a personnel burden on health care providers. Here, we use data from a large cohort of ESUS patients to systematically analyze the effort of ICM remote monitoring for AF diagnosis and the strain on health care providers.

**Methods and results:**

From a prospective, single-center, observational ESUS registry, we analyzed all ICM-equipped patients post-ESUS (n = 172) between January 1^st^, 2018, and December 31^st^, 2019. Through January 2^nd^, 2023, 48 patients (27.9%) were diagnosed with AF by ICM remote monitoring. During follow-up, a total of 29,180 remote monitoring episodes were transmitted, of which 17,742 were alarms for AF. A systematic estimation of workload revealed that on average, 20.3 trained physician workhours are required to diagnose one patient with AF.

**Conclusion:**

ICM remote monitoring is useful to diagnose AF in cohort of post-ESUS patients. However, the number of ICM alarms is high, even in a cohort at known high risk of AF and in whom AF detection is therapeutically consequential. Improved automated event classification, clear recommendations for ICM interrogation after AF diagnosis, and a careful patient selection for ICM monitoring are warranted.

## Introduction

Embolic strokes of undetermined source (ESUS) resemble a subset of strokes for which no underlying cause is found despite a defined diagnostic workup [1]. A diagnosis of ESUS applies to about 20% of all strokes in clinical routine [2]. Diagnostic management includes the search for undetected atrial fibrillation (AF) as a possible underlying cause [3, 4]. AF is found in up to 25% of ESUS patients through intensified rhythm monitoring using implantable cardiac monitors (ICM) and remote monitoring [5]. Importantly, AF detection following ESUS typically results in a clinically relevant change in antithrombotic regimen, from anti-platelet therapy to oral anticoagulation [6]. Yet, implementation of oral anticoagulation for all ESUS patients irrespective of an AF diagnosis has failed to show a clinical benefit [7, 8]. The search for AF and the therapeutic change upon its detection hence remains a key challenge [9].

Clear recommendations for the mode and duration of rhythm monitoring are missing but AF detection rates increase with the duration of monitoring [9]. ICMs offer the longest monitoring duration and in combination with remote monitoring may hence be considered the most suitable means of rhythm assessment after ESUS. However, implementation of ICM remote monitoring into daily routine remains a challenge. ICM implantation is both invasive and particularly costly. In addition, it also imposes a substantial personnel burden onto clinical practice for the review of monitoring alarms. Despite their decidedly diagnostic intention compared to pacemakers and implantable cardioverter defibrillators, ICMs appear to be particularly work intense. ICMs implanted for various cardiologic pathologies have recently been criticized for resulting in a substantial amount of recording transmissions requiring individual assessment by trained physicians [10].

However, analyses of ICM remote monitoring in a high-risk post-ESUS cohort are missing. Here, we analyzed data from a large, investigator-initiated, single-center, observational ESUS registry with a relevant subset of participants equipped with ICMs for rhythm monitoring [11]. We aimed to analyze the diagnostic workload of ICM remote monitoring required to diagnose unrecognized AF. We illustrate impediments and challenges of applied ICM remote monitoring in a high-risk cohort that need to be addressed to facilitate widespread adoption in daily practice.

## Methods

We investigate data of patients implanted with an ICM in our ESUS registry. This registry is an investigator-initiated, single-center cohort that systematically included patients after ESUS between 1^st^ January 2018 and 31^st^ December 2019 and has been reported previously [11, 12]. The study was approved by the Ethics Committee at the LMU, Munich, Germany (project number 17–685) and performed according to current guidelines and regulations.

### Inclusion and exclusion criteria

**Fig 1:**
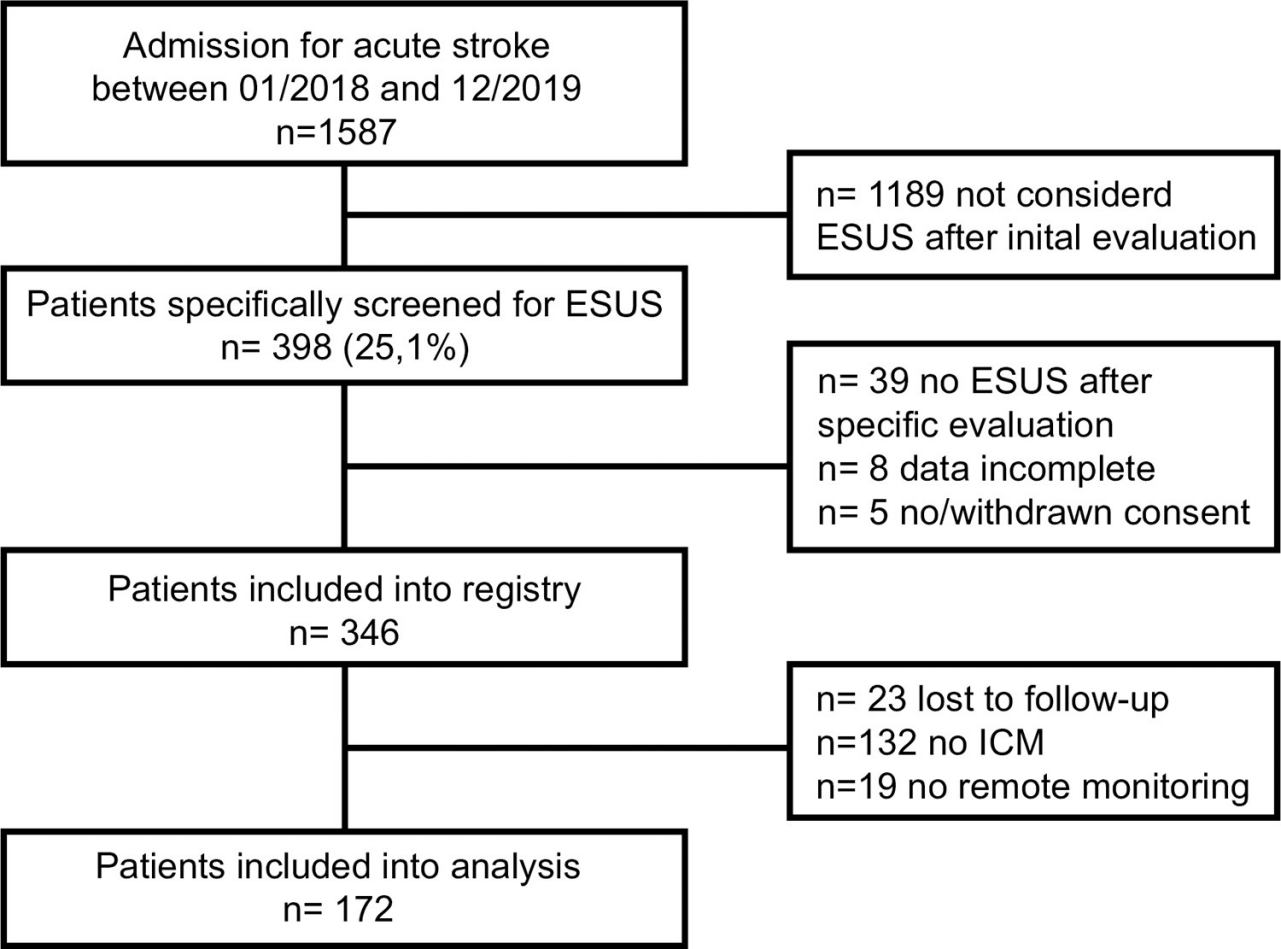
Study flow chart. All patients admitted to the stroke unit for cerebral ischemia were screened for registry enrolment. Patients were excluded in case of non-ESUS stroke etiology. For the present analysis, we further excluded patients who did not receive ICM remote monitoring and those without follow-up information. We thus included 172 patients for analysis.

We included all patients ≥18 years that suffered an ESUS according to current diagnostic guidelines [1], were treated at the LMU University Hospital, Munich, Germany, and provided written informed consent to study participation. ICM implantation was intended for all patients irrespective of age as part of routine clinical care. However, we did not pursue ICM implantation in case of a patient´s objection to implantation, anticipated severely poor neurologic outcome, life expectancy <3 months, and impossibility to enroll into remote monitoring. For our analysis, we excluded all patients that were lost to clinical follow-up and did not receive ICM remote monitoring (for details see **Fig 1**).

### ICM implantation and remote monitoring

All patients underwent ECG monitoring for at least 72h on the stroke unit to rule out prevalent AF prior to ICM implantation. For homogeneity of study results, we relied on a single ICM manufacturer and its pertinent remote monitoring system (Medtronic Reveal Linq ICM®; Medtronic Care Link® home monitoring system, Medtronic, Meerbusch, Germany). All patients received detailed introduction on remote monitoring by a study physician following ICM implantation.

### Clinical endpoint and workload analysis

The clinical outcome was the occurrence of AF of at least 30 second duration, diagnosed by ICM remote monitoring [9]. In case of AF, patients were contacted, and oral anticoagulation was subsequently recommended adhering to guideline recommendations.

To analyze the workload of remote monitoring leading to an AF diagnosis, we summarized all alarms triggered by remote monitoring from the day of implantation until January 2^nd^, 2023. Alarms were automatically categorized by the remote monitoring system into relevant subgroups by presumed underlying causes. This included possible AF, tachycardia, bradycardia, asystole, and symptomatic episodes triggered by the patient. Alarms for AF were further split into (a) a correct first documentation of AF, (b) false positive alarms upon review, which were recorded prior to the correct diagnosis of AF, and (c) any correct or false positive alarm for AF generated after the first correct diagnosis of AF, not implicating further changes to the antithrombotic regimen, but potentially implicating further therapeutic changes in rhythm or rate control.

### Follow up

Remote monitoring of all patients was evaluated every weekday. In case of detection of medically relevant episodes, patients were contacted by phone. This included newly diagnosed AF, relevant bradycardia or atrioventricular block, and supraventricular or non-sustained ventricular tachycardia. In addition, patients were scheduled for in-person follow-up twice a year. All episodes were reviewed and adjudicated by trained physicians. We included all episodes between January 1^st^, 2018 and January 2^nd^, 2023. The individual patient follow-up started with ICM implantation and ended with ICM explantation or administratively on January 2^nd^, 2023.

### Statistical analysis

Continuous values are shown as mean ± standard or median and interquartile range (IQR) as appropriate. Discrete values are presented as absolute and relative frequencies. Comparisons were made by two-sided t-tests and Wilcoxon signed-rank tests for continuous and by Fisher’s exact tests for discrete values as appropriate. Significance was assumed for two-sided p-values <0.05. Statistical analyses were performed using R (version 3.6.1, The R Foundation for Statistical Computing, Vienna, Austria) embedded in R-Studio (version 1.2.1335, Integrated Development for R, RStudio, Boston, MA).

### Workload analysis per patient

We estimated the number of episodes to be assessed when monitoring one patient for one year. For this, we used the total number of episodes recorded within the median follow up time for all 172 patients and derived the total number of episodes per year. We subsequently divided the total number of episodes per year by the number of patients under observation. The number of telephone calls and on-site visits per patient per year were derived accordingly. The number of patients to be monitored to diagnose one patient with AF resulted from the inverse of the incidence of AF in our cohort within the first year of monitoring.

### Workload analysis per 100 patients

To illustrate the workload for physicians monitoring 100 patients for one year, we calculated the total episodes to be assessed from the mean number of episodes per patient and year. Telephone calls and on-site visits were subsequently estimated. The estimated number of AF cases among 100 patients monitored for one year was calculated using the incidence of AF within the cohort during the first year of monitoring.

We assumed an average of 2 minutes required for an experienced physician to assess, interpret, and inform about one episode of remote monitoring. While the assessment of AF episodes leading to the final documentation of AF usually take longer than 2 minutes, assessment of known AF episodes after the initiation of oral anticoagulation are processed faster. The average time of 2 minutes was estimated from our center’s long-standing experience in daily remote monitoring. Applying this amount of time, we calculated the total time required to assess all triggered alarms during the entire follow-up duration and per year, respectively.

## Results

### Study population

From January 2018 to December 2019, we prospectively enrolled 346 ESUS patients into the ESUS registry. We excluded 23 patients who were lost to follow-up, 132 who did not receive an ICM, and 19 who could not be enrolled into the remote monitoring system. Hence, 172 patients were available for analysis (**Fig 1**).

Our cohort had a mean age of 64.2±11.7 years and was predominantly male (68.6%). The prevalence of cardiovascular risk factors including hypertension (68.0%), hypercholesterolemia (40.7%), diabetes (18.6%), or smoking (34.3%) was high (**Table 1**). The majority of the cohort (n = 138, 80.2%) had low HAVOC scores suggesting an overall lower AF risk after ESUS [13].

**Table 1:** Baseline characteristics.

	Total	AF during FU	no AF during FU	p
	(n = 172)	(n = 48)	(n = 124)	
**Age [years]**	64.2±11.7	69.8±10.7	62.0±11.4	<0.001*
**Male sex [n (%)]**	118 (68.6%)	31 (64.6%)	87 (70.2%)	0.583
**BMI [kg/m** ^**2**^ **]**	26.6±4.1	26.8±3.9	26.5±4.1	0.606
**Hypertension [n (%)]**	117 (68.0%)	35 (72.9%)	82 (66.1%)	0.468
**Hypercholesterolaemia [n (%)]**	70 (40.7%)	23 (47.9%)	47 (37.9%)	0.468
**Diabetes [n (%)]**	32 (18.6%)	11 (22.9%)	21 (16.9%)	0.387
**Ever Smoker [n (%)]**	59 (34.3%)	12 (25.0%)	47 (37.9%)	0.152
**Neurologic status**				
**NIHSS admission [median (25th;75th)]**	2 (1;3.25)	1 (0;2.25)	2 (1;4)	0.016*
**NIHSS discharge [median (25th;75th)]**	1 (0;2)	0 (0;1)	1 (0;2)	0.039*
**mRS discharge [median (25th;75th)]**	1 (0;2)	1 (0;1)	1 (0;2)	0.191
**Medication**				
**Antiplatelet therapy at discharge [n (%)]**	158 (91.9%)	45 (93.8%)	113 (91.1%)	0.760
**OAC at discharge [n (%)]**	16 (9.3%)	4 (8.3%)	12 (9.7%)	1.00
**ACE/ARB enrolment [n (%)]**	79 (45.9%)	27 (56.3%)	52 (41.9%)	0.124
**Statin enrolment [n (%)]**	55 (32.0%)	21 (43.8%)	34 (27.4%)	0.046*
**BB enrolment [n (%)]**	49 (28.5%)	19 (39.6%)	30 (24.2%)	0.059
**Diuretics enrolment [n (%)]**	34 (19.8%)	10 (20.8%)	24 (19.4%)	0.833
**Scores**				
**CHADS-VASc [median (25th;75th)]**	4 (3;5)	5 (3;6)	4 (3;5)	0.002*
**Rhythm Irregularity burden [median (25th;75th)]**	1.0 (1.0;1.1)	1.2 (1.0;1.9)	1.0 (1.0;1.0)	<0.001*
**HAVOC low risk [n (%)]**	138 (80.2%)	32 (66.7%)	106 (85.5%)	0.009*
**HAVOC intermediate risk [n (%)]**	31 (18.0%)	14 (29.2%)	17 (13.7%)	0.026*
**HAVOC high risk [n (%)]**	3 (1.7%)	2 (4.2%)	1 (0.8%)	0.189
**Echocardiography**				
**EF [%]**	59.2±5.1	59.3±4.5	59.2±5.4	0.940
**LA diameter [mm]**	33.6±6.3	36.4±6.3	32.5±6.0	0.003*
**Relevant PFO [n (%)]**	27 (33.3%)	8 (16.7%)	19 (15.3%)	0.127
**Laboratory**				
**NT-proBNP [pg/ml]**	122 (64.4;305.5)	153 (83.4;562.5)	110 (57.1;253.5)	0.039*
**HbA1c [%]**	5.7 (5.4;6.1)	5.8 (5.5;6.5)	5.7 (5.4;6.1)	0.098

Abbreviations: AF–atrial fibrillation, FU–Follow-up. BMI–Body Mass Index, NIHSS–National Institutes of Health Stroke Scale, mRS–modified Rankin Scale, OAC–Oral Anticoagulation, ACE–ACE Inhibitor, ARB–Angiotensin II Receptor Blocker, BB–Betablocker, EF–Ejection Fraction, LA–left atrial, PFO–Patent foramen ovale

### Clinical endpoint

Of 172 patients in our cohort, 48 patients (27.9%) were diagnosed with AF by ICM analysis until January 2^nd^, 2023. The median duration of follow-up was 1,323 days (1,204;1,481). Patients who developed AF were significantly older (69.8±10.7 years vs. 62.0±11.4 years, p<0.001). Eleven patients were <60 years, whereas 37 patients were ≥60 years at AF documentation. AF patients also had higher CHA_2_DS_2_-VASc scores (5 (3;6) vs. 4 (3;5), p = 0.002), and had larger left atrial diameters (36.4±6.3mm vs. 32.5±6.0mm, p = 0.003), both of which are known risk factors for AF (**Table 1**). Interestingly, patients who developed AF showed significantly lower NIHSS scores both at admission and at discharge. Finally, the recently published rhythm irregularity burden was significantly higher for patients with AF (1.2 (1.0;1.9) vs 1.0 (1.0;1.0), p <0.001) (12).

The median duration from ICM implantation until AF diagnosis was 189 (38.5;642.8) days (range: 0 to 1095 days). Most patients (n = 32, 66.7%) were diagnosed with AF in the first 12 months of monitoring. Of particular note, 8 patients developed AF after interventional closure of a patent foramen ovale. Patients with AF after interventional closure were all <60 years and had a CHA_2_D_2_S-VASc Score significantly lower than AF patients who did not receive an intervention (3 (3;3) vs. 5 (4;6), p<0.001).

### Remote monitoring in clinical practice

**Fig 2:**
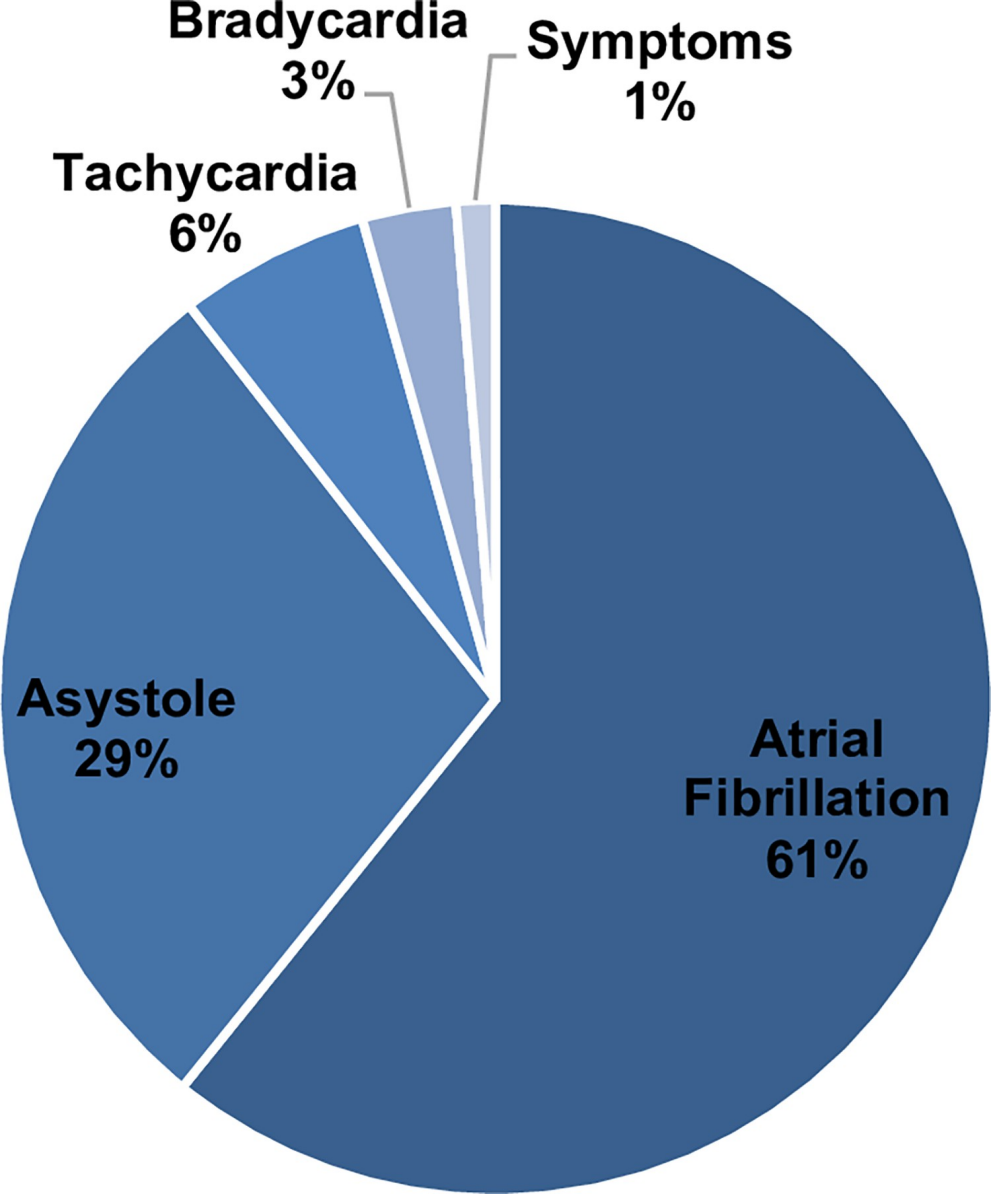
Illustration of alarm subtypes. The pie chart illustrates the distribution of alarm subtypes generated by ICM remote monitoring. In total, 29,180 episodes were assessed.

All 172 patients were enrolled into remote monitoring. Of these, 82.6% of participants (n = 142) successfully installed their devices at home and regularly transmitted episodes. Over the entire follow-up period (median 1,323 days (1,204;1,481)), a total of 29,180 episodes were transmitted. All episodes were individually reviewed by trained physicians. Of all recorded episodes, 17.742 (60.8%) alarms were triggered for suspected AF. Of the AF alarms, 1,121 (6.3%) were classified as false positive and occurred in patients prior to an adjudicated diagnosis of AF. 48 AF alarms were classified as correct and lead to the clinical diagnosis of AF after ESUS. The remaining 16,573 AF-alarms occurred after a correctly adjudicated diagnosis of AF. These alarms were routinely assessed to evaluate AF burden, discuss options of rhythm control, or optimize medical frequency control. **Fig 2** illustrates the distribution of alarms into subgroups adjudicated by the remote monitoring system. Beyond AF alarms, 8,366 alarms were recorded due to asystole. This included short, asymptomatic pauses, episodes of undersensing, and implantation artefacts. Alarms due to tachycardia (n = 1,814, 6.2%), bradycardia (n = 887, 3.0%) or patient triggered symptoms (n = 371, 1.3%) occurred less frequently.

In addition to the regular assessment of episodes transmitted by remote monitoring, we conducted a total of 581 on site visits and 222 telephone calls to inquire about symptoms, to inform about the diagnosis of AF or other clinically relevant finings, and to initiate a change in antithrombotic regimen from antiplatelet therapy to oral anticoagulation if indicated. Such a change in medical management occurred in 45 (93.8%) patients diagnosed with AF. Of the remaining three patients newly diagnosed with AF, two only had short AF episodes during interventional closure of a patent foramen ovale or after cardiac surgery, respectively. In both cases, extended rhythm monitoring was conducted and no change in medical treatment was initiated. The third patient was contacted but opted to defer a decision to change to anticoagulant treatment.

Four patients died while under remote monitoring, and six patients received a pacemaker with concomitant ICM explantation. 36 ICMs were explanted at the end of battery life or per patient preference after AF had been diagnosed. Further 41 ICMs had notified EOS (end of service) or RRT (recommended replacement time) and follow-up was subsequently terminated. Overall, 85 ICMs (49.4%) were still actively monitoring at the end of administrative follow-up on January 2^nd^, 2023.

### Assessment of the diagnostic burden for ICM analysis in ESUS patients at risk for AF

**Fig 3:**
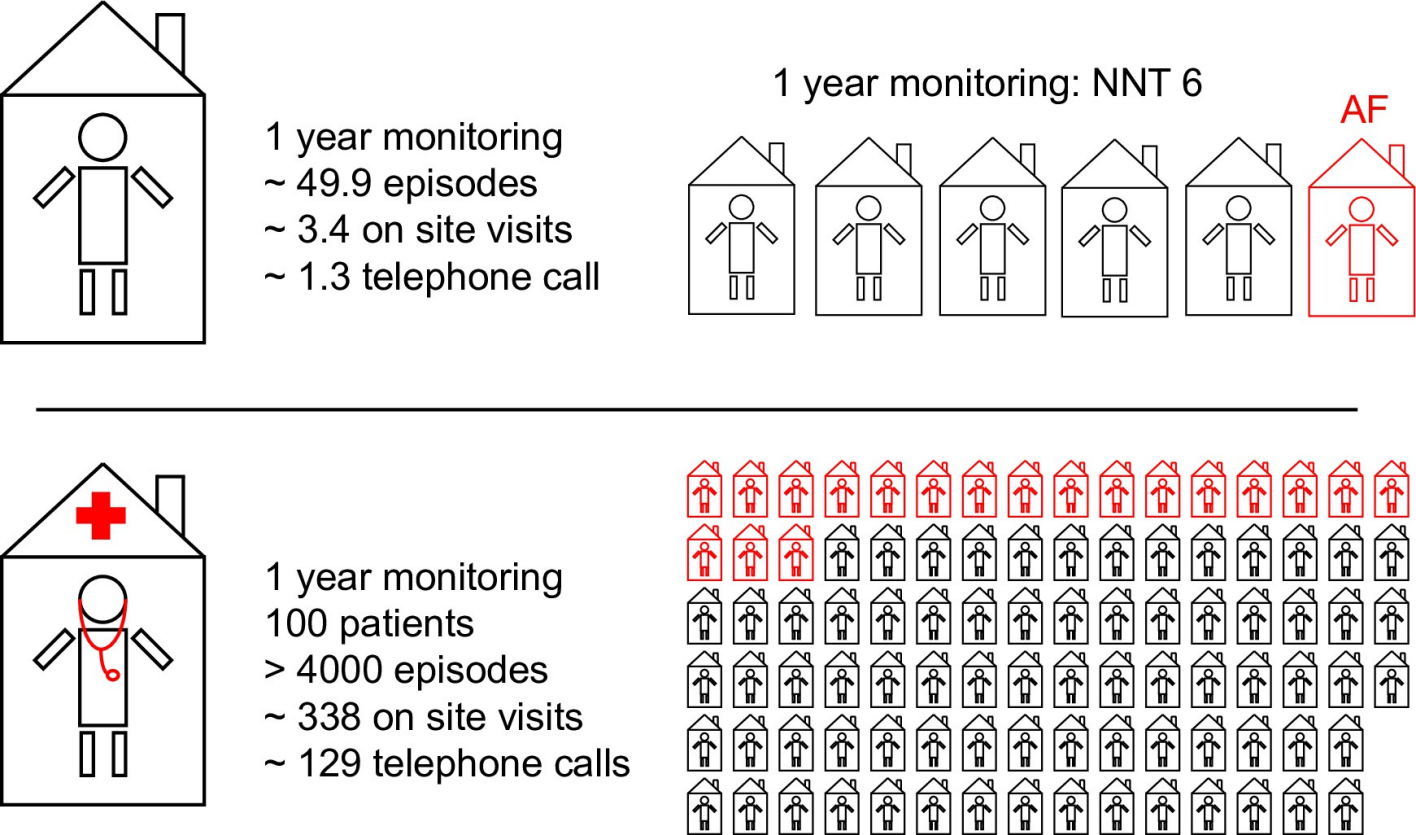
Remote monitoring in clinical routine. The figure visualizes the workload to diagnose AF by ICM remote monitoring, both from the individual patients’ perspective (upper panel) and from the physician’s perspective (lower panel). Above, a single patient generates an average of 49.9 episodes per year, reports to 3.4 on-site visits per year, and responds to 1.3 telephone calls per year. In contrast, a team of physicians coordinating a remote monitoring system with 100 enrolled patients will need to adjudicate >4000 episodes per year, will need to conduct an average of 338 on-site visits per year, and will perform an average of 129 telephone calls per year.

We estimated the burden to conduct ICM remote monitoring in ESUS patient at risk for AF in clinical practice by assessing the average workload per individual patient. Monitoring the ICM data of one patient for the duration of one year resulted in a mean of 49.9±228 episodes for assessment by an experienced physician. On top, 3.4±2.6 on-site visits and 1.3±1.8 telephone calls had to be performed. Based on our data, six patients had to be monitored by ICM to diagnose one patient with AF within the first year of monitoring (**Fig 3**). In consequence, for a telemedicine unit following 100 ESUS patients at risk for AF by remote monitoring for one year would result in >4000 recorded episodes for individual assessment. In addition, 338 on-site visits would have to be scheduled, and 129 additional telephone calls would be required. Of 100 patients, 19 patients would develop AF within one year (**Fig 3**).

We assumed that evaluation of one ICM-recorded episode, contacting the patient for information if necessary, and eventually initiating a change in medical treatment requires two minutes on average. As a result, the evaluation of all 29,180 episodes resulted in an estimated temporal effort of 973h of remote monitoring work overall and a median time of 268h per year. Focusing on the diagnosis of AF, an average of 20.3h hours of working time by a physician would have been required to diagnose a single patient with AF.

## Discussion

This systematic analysis of intensified rhythm monitoring for patients post ESUS shows how ICM remote monitoring is a potent telemedicine tool to diagnose AF. With 27.9% ICM-detected AF, our study validates the clinical benefit of this intensified monitoring approach in a high-risk cohort [5]. However, ICM remote monitoring also imposes a substantial workload on health care providers. We estimate an average of 20.3 hours of working time to diagnose a single patient with AF.

Despite this intensive workload the use of home monitoring in a cohort at high risk for AF is often discussed as exceptionally useful as detection of AF has a direct impact on antithrombotic management, with initiation of oral anticoagulation in a majority of patients [9]. In contrast to non-ECG-based monitoring approaches, initiation of oral anticoagulation can be started immediately after AF detection. Intermittent as opposed to ICM-based continuous rhythm monitoring potentially delays diagnosis by missing non-persistent, asymptomatic AF episodes. Furthermore, the potential use of ICM-monitoring is not limited to ESUS patients as other stroke subtypes including patients with small vessel disease and large-artery atherosclerosis also show a high AF-incidence as proposed by Bernstein et al. [14]. However, systematic analyses are missing that show the benefit of an ICM-based intensified rhythm monitoring with telemedical care for the reduction of recurrent strokes. While multiple studies in non-stroke cohorts have failed to show a benefit of intensified AF screening to reduce clinically relevant incidental strokes [15, 16], future studies are needed to evaluate this approach in a high-risk ESUS cohort and to directly compare its effectiveness to non-invasive monitoring devices in the light of the high workload needed.

Most recently, the need to commence anticoagulation following the identification of device-detected atrial high rate episodes (AHRE) as studied in NOAH-AFNET6 [17] or subclinical AF as studied in ARTESIA [18] has been challenged. Importantly, neither of the two trials investigated ESUS or stroke patients more generally, and hence such patients were underrepresented in both studies. In addition, both trials enrolled patients where AHRE and subclinical AF, respectively, where the result of opportunistic screening, whereas in our ESUS cohort, monitoring was done with the deliberate intention to search for presumable underlying causes of the cerebral ischemia. Given that both NOAH-AFNET6 and ARTESIA demonstrated that anticoagulation may also result in harm, we suggest that also in ESUS patients with ICM-detected AF the guideline-recommended initiation of anticoagulation is carefully evaluated. However, future studies need to specifically address this important problem until the generalizability of the NOAH-AFNET6 and ARTSIA results can be adjudicated”.

Clinical practice is challenged by multiple factors. Most importantly, although in our high-risk cohort every sixth patient was diagnosed with AF, remote monitoring accumulated >29,000 episodes for individual review by experienced physicians. This is well in line with previous studies illustrating the exceptionally high amount of alarms of ICMs in comparison to pacemakers or implantable cardioverter-defibrillators [10]. While O’Shea et al. used data for ICM monitoring for multiple underlying diseases including syncope or bradycardias, our present study illustrates the burden of alarms in a specified cohort post ESUS at high risk for AF. Furthermore, our analysis includes a long follow-up time with a majority of ICMs being analyzed for their entire lifetime.

Most alarms were triggered for AF. Of these, 6.3% were false positive AF alerts prior to an adjudicated correct diagnosis of AF. Previous studies have also suggested alarms for AF or atrial tachycardias being most frequent and even found a higher burden of 74.2% false positive alarms for this alarm entity [19]. This difference may presumably be explained as we only regarded episodes as false positive before a first, correct diagnosis of AF. All subsequent alarms for AF, whether false positive or correct, did not lead to changes in antithrombotic regimens as oral anticoagulation had already been started. However, these alarms were nevertheless important as patients were contacted in case of episodes of tachycardia to optimize rate control strategies and evaluate the indication of rhythm control. Prolonged monitoring has hence the capability to improve treatment of patients with documented AF. Yet, the large number of AF episodes accumulating over time still imposes a significant work burden on health care providers. Clear recommendations in which clinical situations ICM monitoring should be deescalated following a successful AF detection are missing but are critically needed.

Recognizing the need for better patient selection prior to ICM implantation the European Society of Cardiology has suggested the use of risk scores to select patients with cryptogenic stroke at high-risk of AF prior to ICM-implantation [20, 21]. One example is the HAVOC score introduced by Elkind and colleagues in 2021 [13]. In our cohort, 138 patients (80.2%) had low HAVOC scores, 31 (18.0%) had intermediate HAVOC scores and 3 (1.7%) had high HAVOC scores. Thus, most of our cohort were at lower AF risk, which emphasizes that these patients may not have been ideal candidates for invasive continuous monitoring. Patients with lower HAVOC scores additionally developed AF significantly less frequent underlining the power of this score to identify patients at lower AF risk. However, also the majority of patients with AF during follow-up were categorized as low risk by the HAVOC score. The score may hence serve as an additional but not as the sole criterion for decision-making. Further investigations in larger cohorts are needed to better evaluate the usability of the score in clinical practice.

Alarms due to asystole represented the second most frequent subtype confirming previous analyses [19]. However, most asystole episodes upon review were caused by undersensing. Improvement of hardware and new software-based automated event classification is required to improve correct alarm classification and thereby reduce the workload for physician review of episodes.

Importantly, six patients were diagnosed with relevant bradycardias and received a pacemaker with concomitant ICM explantation although the initial intention for ICM implantation was the search for AF instead of symptomatic bradycardias or syncopes. This result is well in line with previous investigations of relevant subsidiary findings on ICM remote monitoring. A study by Bauer et. al. used ICMs in patients after myocardial infarction with moderately reduced left ventricular ejection fraction, primarily intended for the identification of ventricular arrhythmias [22]. However, the study also documented a high rate of AF and atrioventricular block resulting in permanent pacemaker implantation. Together with our results, this underlies that ICM implantation in high-risk cohorts post myocardial infarction or post stroke may be useful beyond the initial rationale for monitoring. To that end, the duration of ICM monitoring should be exploited a long as possible in principle. However, such prolonged monitoring meanwhile generates numerous alarms. Hence, clear recommendations by alarm subtype should be developed depending on the individual clinical background to reduce the relevant workload for health care providers.

Only 82.6% of patients successfully installed the remote monitoring system at home and transmitted episodes regularly thereafter. Almost one fifth of patients did not transmit although every patient received a personal introduction by an experienced physician on top of the installation instructions by the manufacturer. Modern equipment with more convenient handling is required to increase the number of successful regular transmissions by patients. This is of particular importance in a post ESUS cohort, where intensified rhythm monitoring is applied in oftentimes functionally disabled individuals. In turn, future patients may be more intuitively accustomed to using remote monitoring equipment, which may as well improve the rate of successful transmissions.

Some limitations merit further discussion. This single-center registry prospectively followed patients post ESUS. However, larger multicenter observations of remote monitoring are needed to estimate the workload independent of the processes of our center and to evaluate possible differences across various health care systems. Furthermore, our analysis included 17.4% of patients not installing remote monitoring and several patients not attending their scheduled on-site visits. To overcome these challenges remains critical in daily routine but in turn reflects the real-world scenario of our cohort. Furthermore, the selection of patients for ICM implantation remained a clinical decision of the treating neurologist based on the clinical prognosis, so that a selection bias cannot be completely ruled out”.

In conclusion, ICM remote monitoring is a potent tool to detect AF in a high-risk ESUS cohort. ICM remote monitoring has the potential to reclassify ESUS and subsequently optimize antithrombotic treatment towards guideline-indicated oral anticoagulation. However, our analysis demonstrates that relevant effort is required to implement and maintain timely adjudication of events for the benefit of the patient. Optimized automated event classification and careful indication and patient selection appear warranted.
